# Distinctive biochemistry profiles associated with hyperuricemia between Tibetans and Hans in China

**DOI:** 10.3389/fendo.2023.1229659

**Published:** 2023-11-28

**Authors:** Xue-Wen Ren, Kang Chen, Jue Wu, Zhang-Lin Yang, Tao Ji, Qing-Hong Zhang

**Affiliations:** ^1^ Department of Emergency Medicine, First Medical Center of General Hospital of Chinese People’s Liberation Army, Beijing, China; ^2^ Department of Endocrinology, First Medical Center of General Hospital of Chinese People’s Liberation Army, Beijing, China; ^3^ Translational Medicine Research Center, General Hospital of Chinese People’s Liberation Army, Beijing, China; ^4^ Trauma Repair and Tissue Regeneration Center, Department of Medical Innovation Study, General Hospital of Chinese People’s Liberation Army, Beijing, China

**Keywords:** Tibetan, hyperuricemia, hypoxia, glycolysis, aminotransferase, lipid metabolism, red blood cell

## Abstract

**Purpose:**

We sought to identify distinct risk factors for hyperuricemia in native Tibetan and immigrant Han populations in Tibet, China.

**Methods:**

Three cohorts of male participants aged between 20 and 40 years were enrolled in this study. Biochemical parameters including serum uric acid (UA), fasting plasma glucose, insulin, lactate dehydrogenase (LDH), thyroxin, blood cell count, aminotransferase, and lipid profiles were analyzed. The association of risk factors with UA levels was evaluated using a multivariable line regression model. The effect of UA level on the biochemical parameters between the Hans and Tibetans was evaluated by two-way ANOVA.

**Results:**

The prevalence of hyperuricemia (≥420 μmol/L) was 24.8% (62/250) in the Hans, similar to 23.8% (29/136) in the Tibetans. In the regression analysis, the risk factors that were significantly associated with UA in Hans did not apply to Tibetans. Tibetans had higher fasting insulin (*P*<0.05) and LDH (*P*<0.01) levels, in contrast with lower levels of triglycerides (*P*<0.05), total cholesterol (*P*<0.01), and low-density lipoprotein-cholesterol (*P*<0.01) than Hans in normal UA populations. Biochemistry analysis revealed lower albumin levels (*P*<0.001) and higher levels of all aminotransaminase and especially alkaline phosphatase (*P*<0.01) in Tibetans than in Hans in both populations. Compared with Hans, Tibetans had lower serum levels of urea, creatinine, and electrolytes in the normal UA population, which were further exacerbated in the high UA population. Tibetans had comparable white blood cell counts as Hans in both normal and high UA populations. In contrast, the red blood cell count and hemoglobin concentration were much lower in Tibetans than in Hans under high UA conditions.

**Conclusions:**

The distinctive biochemistry between Tibetans and Hans may underlie the different etiologies of hyperuricemia in Tibet, China.

## Graphical Abstract

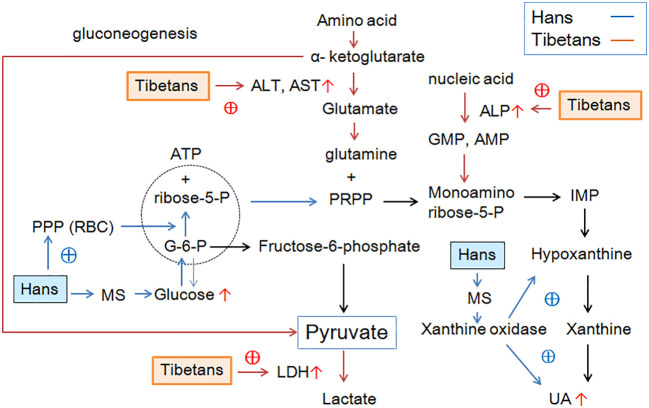

## Introduction

Uric acid (UA) is the end product of purine metabolism and plays a key role in the pathogenesis of gout and other diseases including diabetes, hypertension, and chronic kidney disease. In addition, hyperuricemia is significantly associated with the prevalence of metabolic syndrome (MS) (1, 2). The prevailing view is that the prevalence of hyperuricemia in Chinese Tibet is much higher than that in other parts of China, except for one study that reported a relatively lower prevalence of gout (0.30%) and hyperuricemia (1.83%) in Naqu, Chinese Tibet (3). However, the prevalence of hyperuricemia in immigrants in Chinese Tibetan region was 37.2% in Ganzi (4), 54.2% in Shannan (5), and 40.7% in general Tibetans region (1). In contrast, the prevalence of hyperuricemia was 13.3% in inland China (6), 6.4% in Chinese middle-aged and older adults (7), 10.2% in Chinese rural areas (8), and 15.4% in Hans from northwest China (9). In two Italian population, the prevalence of hyperuricemia (>7.0 mg/dL) in men was 12.9% (56/435) (10) and 7.3%, respectively (11). Regarding ethnicity, the prevalence of hyperuricemia was much higher in Tibetans than in Hans (58.8% *vs.* 28.4%, *P*<0.001) in the same Tibetan region (4). Several factors have been suggested to contribute to the high prevalence of hyperuricemia in the Tibetan Plateau, including MS components (1), ethnicity, dietary habits, hypoxic environment, and gene polymorphisms (12).

Nowadays, with the development of economy and tourism, more inlanders would settle in Chinese Tibet area. The above surveys raised a great concern that Han inlanders accumulated increasing levels of UA if they assimilated into the high altitude as Tibetan highlanders. It has been reported that the clinical indices of Hans were increasingly similar to those of Tibetans with their plateau living (13). Tibetans have undergone natural selection toward a phenotype of exceptional tolerance to hypobaric hypoxia compared to non-Tibetans living at the same altitude (14). Adaptation to acute hypoxia consists of a variety of physiological, metabolic, and molecular changes, such as increased uptake and oxidation of circulatory glucose during exercise (15), increased circulating nitric oxide metabolites (16) to increase vasodilatation and blood flow (16), a shift from aerobic to anaerobic metabolism that favors glycolytic over fatty acid energy supply (17, 18), an increase in muscle glucose toward the pentose phosphate pathway (PPP) (19) to improve muscle energetic performance (18), and protection against oxidative stress (18) with decreased hemoglobin concentration [Hb] (20). The advantageous haplotype of peroxisome proliferator-activated receptor alpha (PPARA) is associated with a lower capacity for fatty acid oxidation in skeletal muscle in Tibetans (21) and in high-altitude Sherpas (18). Adaptation to chronic hypoxia also involves relative hypometabolism in the brain to minimize the impact of oxygen limitation (22). Therefore, it is necessary to elucidate the different adaptive mechanisms underlying hyperuricemia between immigrant Hans and native Tibetans on the Chinese Tibetan Plateau.

A high-altitude environment features sustained hypobaric hypoxia (23). Acute hypoxia directly enhances UA production and secretion. The phosphorylation of critical enzymes for UA production, xanthine dehydrogenase/xanthine oxidase (XO), was greatly increased (50-fold) in response to acute hypoxia in rat pulmonary microvascular endothelial cells (24). Adipose tissue has abundant expression and activity of xanthine oxidoreductase (XOR). Adipocyte UA secretion increases under hypoxia (25). Furthermore, it was demonstrated that hypoxia diminishes adenosine triphosphate (ATP) utilization by downregulating the activity of Na-K-ATPase in proximal renal tubular epithelial cells (26), limiting renal filtration and excretion ability in rats (27). These findings emphasize that exposure to a hypobaric hypoxic environment may play a crucial role in the pathogenesis of hyperuricemia in the Tibetan Plateau. The discrepancy in evolutionary adaptation toward the cruel environment between the Hans and Tibetans may underlie the unique etiology of hyperuricemia in Tibetans.

However, most of the related studies in Tibetans were on middle-aged or old populations who had already assimilated to high altitudes for many years and may have developed other confounding diseases. Considering that hypoxic research on healthy individuals at high altitudes may be translated into hypoxemic critically ill patients in a hospital setting (14), we carried out a thorough survey of three cohorts of populations aged 20-40 years and compared the biochemical profiles between native Tibetans and immigrant Hans on the Chinese Tibetan Plateau. We aimed to identify the distinctive mechanism underlying the high incidence of hyperuricemia in highland Tibetans and inland Hans acclimatizing to high altitudes.

## Methods

### Study participants

All study protocols were approved by the ethics committee of the Chinese PLA General Hospital (approval identifier S2021-016-01) and were in accordance with established national and institutional ethical guidelines. The study was clearly described to all participants who signed informed consent forms before the collection of blood and personal information. Healthy adults aged 18-60 years old who had lived in the Tibetan region for > 1 year were included in the study. Participants with major operation, tumor, severe lung, heart, digestive, or endocrine diseases were excluded.

### Survey method and data collection

A comprehensive questionnaire, including questions on demographics, medical history, and lifestyle risk factors, was administered by the staff at local health stations according to a standard protocol. Systolic blood pressure (SBP) and diastolic blood pressure (DBP) were measured using a standardized automatic electronic sphygmomanometer (Omron HEM-770A). Body weight (BW) and height were measured, and body mass index (BMI) was calculated as weight/height2 (kg/m2).

### Sample collection

From April 2018 to October 2022, we recruited three groups of healthy male adults (18-60 years old) from both the Han and Tibetan populations in the Chinese Tibetan region (**Table 1**). Routine physical examinations were conducted in three counties at three altitudes: Lhasa (altitude: 3670-3835 m), Nyingchi (altitude: approximately 2900 m), and Naqu (altitude:4298-4352 m). Group A (n=149) covered suburban Lhasa and Nyingchi, including teachers, students, soldiers, workers, and a few farmers. A standard questionnaire, including information on diet, exercise, altitude, and smoking, was administered. Plasma samples from Group A transported and subjected to thorough biochemical analysis in Beijing, China. Group B (n=226) covered only urban Lhasa and mainly consisted of civil servants, with a few items of biochemistry and routine blood cell counts analyzed immediately on site. Group C (n=111) recruited young adults in Tibetan Naqu, with routine blood cell counts and a few biochemical parameters analyzed immediately at the local station (**Table 1**). Sampling was conducted between April and October to avoid seasonal variation. To avoid possible systematic errors, doctors (XWR) instructed local medical staff and monitored the data collection and detection procedures.

**Table 1:** Charactteristic of the three populations in high-altitude regions of Tibet, China.

Population	Time	Demography	Ethnicity		*P* value
			Hans	Tibetan	
Group A (n=149)	Apr, 2018	n	80	69	
		Age (year), median (IQR)	25 (21-30)	29 (18-41)	0.032
		Location	Lshsa and Nyingchi,Tibetan		
		Livelihood	teachers, student, soldiers, worker,farmer,herdsman		
		Altitude	3670-3835 m, 2900 m		
		Sample analysis	PLA General Hospital, Beijing		
		Hyperuricemia, n (%)	50 (62.5)	24 (34.8)	
Group B (n=226)	Jan-Dec, 2018	n	70	156	
		Age, median (IQR)	33 (24-47)	42 (33-52)	0.004
		Location	Lshsa,Tibetan		
		Livelihood	civil servant		
		Altitude	3670-3835 m		
		Sample analysis	Lshsa Peoples’ Hospital, Lshsa, Tibet		
		Hyperuricemia, n (%)	31 (44.3)	51 (32.7)	
Group C (n=111)	Oct, 2018	n	46	65	
		Age, median (IQR)	20 (19-22)	19 (18-20)	0.020
		Location	Naqu,Tibetan		
		Livelihood	student,worker,farmer,herdsman		
		Altitude	4298-4352 m		
		Sample analysis	Naqu Peoples’ Hospital, Naqu, Tibet		
		Hyperuricemia, n (%)	16 (34.8)	13 (20.0)	

IQR, interquartile range; PLA, Peoples Republic of Chima.

### Collection of blood and measurements

Fasting venous blood (8 ml) was collected in EDTA-K2 tubes. Hematological parameters were determined immediately using an automated hematology analyzer (Sysmex KX-21, Japan). Blood samples were separated by centrifugation at 4,000 rpm for 10 min within 4 h and then stored at -80°C (Group A) or subjected to automatic analysis of a few biochemical indicators (Hitachi 7180, Japan) immediately in Tibetan Peoples’ Hospital (Group B) or Naqu Peoples’ Hospital (Group C).

Plasma samples in Group A were transported and assayed in the Department of Laboratory Medicine of Chinese PLA General Hospital (Beijing) (Group A), including UA, glucose, lactate dehydrogenase (LDH), albumin, alanine aminotransferase (ALT), aspartate aminotransferase (AST), γ-glutamyl transferase (GGT), total bilirubin (TBIL), and direct bilirubin (DBIL); renal parameters, including creatinine, urea, and cystatin C; and lipid parameters, including triglyceride (TRIG), total cholesterol (TCHOL), high-density lipoprotein cholesterol (HDL-CH), low-density lipoprotein- cholesterol (LDL-CH), and electrolytes, using an automatic biochemical immunity analyzer (Cobas 8000, Roche, USA). The remaining plasma (1.5 ml) was used for hormone assays. The thyroxin concentration was determined using an automated chemiluminescence immunoassay analyzer (ADVIA Centaur^®^XP, Siemens, Germany). Insulin was detected using a chemiluminescence immunoassay analyzer (Cobas e601; Roche, Switzerland). Tissue nonspecific alkaline phosphatase (ALP) was detected using colorimetry (Roche Diagnostic GMBH, Mannheim, Germany).

### Definitions of hyperuricemia

Considering that the males predominate over the females in the incidence of hyperuricemia (1, 4), because of the limitation of the page space and concision of the study, we only present the male data this time. Hyperuricemia in males was defined as a fasting serum UA level≥420 μmol/L according to Chinese guidelines (28).

### Statistical analyses

The normality of the variables was analyzed using the “Shapiro-Wilk test.” The mean and standard deviation were used to describe variables that met the normal distribution, and the median and interquartile (IQR) distance were used to describe variables that did not meet the normal distribution. GraphPad Prism 9.0 (GraphPad Software, USA) was used for data analysis. The two ethnic populations in Group A (Lhasa and Nyingchi) were subjected to multiple line regression analysis to estimate the associations between the major indices and UA levels, including age, altitude, BMI, and biochemical parameters. Where the dependent variable was affected by UA level and ethnicity, the data were analyzed using two-way ANOVA. When the main effect or interaction was significant, *post-hoc* analyses using the Bonferroni correction were performed. Differences between subjects were analyzed using the Chi-squared test or Fisher’s exact test for categorical data.

Group B (Lhasa) was subjected to the calculation of the correlation coefficients (*r*) between the red blood cell (RBC) profile and UA level using Pearson’s analysis. A two-sided *P*<0.05 was considered statistically significant. Multivariate line regression was used to analyze the associations between RBCs indices and UA levels in the two populations.

Group C (Naqu) was used for the comparison of demography, RBCs profile, and blood cell count between Hans and Tibetans. The Mann–Whitney U test or unpaired *t* test was used for intergroup comparisons.

## Results

### Characteristics of the three cohorts of populations

The three groups of populations covered the ages 20-30 (Group A), 30-40 (Group B), and 19-20 (Group C) years old. Tibetans were older than Hans in the former two populations, whereas they were younger than Hans in Group C (**Table 1**).

The survey of Group A was based on a sample drawn from a larger population. We first enrolled 250 Hans and 136 native Tibetans for general physical examinations. The incidence of hyperuricemia was 24.8% in the total Han population (62/250) with a median (IQR) age (years) of 24 (21-28) and 23.8% in the total native Tibetan population (29/136) with a median (IQR) age of 31 (19-46). From the above population, we matched samples with similar ages and backgrounds into Group A for the following thorough biochemistry analysis, in which the Hans (n=85) were still younger than the Tibetans (years) (n=64) [25 (21-30) *vs.* 29 (18-41), *P*=0.032]. However, there was no difference in age between the subgroups (**Table 2**). The median (IQR) length of residence (years) for the Hans in Tibet was similar between the subgroups with high UA and normal UA levels [3.3 (1.3-7.8) *vs.* 3.7 (1.3-6.3), *P*=0.53].

**Table 2:** Comparison of the characteristic between Tibetans and Hans in Tibet, China (Group A).

Variates	Hans			Tibetan			P value (Hans vs.Tibetan)	
	normal (n=30)	high (n=55)	P value	normal (n=37)	high (n=27)	P value	Normal	High
age (year; median, IQR)	23.0 (21.0-35.0)	26.0 (21.0-30.0)	0.11	20.0 (17.0-41.5)	32.0 (25.0-46.0)	0.61	0.09	0.71
Height (cm)	173.5 (168.0-177.0)	173.0 (169.0-176.0)	0.60	168.5(160.0-173.8)	175.0(169.3-180.0)	0.002	0.011	0.31
Body weight (kg)	67.0 (58.8-122.5)	70.0 (62.0-88.5)	0.59	100.0 (92.00-140.0)	90.0 (80.0-103.0)	0.21	0.017	0.11
BMI (median, IQR)	22.50 (20.0-44.0)	23.5 (21.0-29.0)	0.44	37.5(32.0-47.8)	29.5 (25.3-35.8)	0.13	0.014	0.10
Length of stay in Tibetan	3.7 (1.3-6.3)	3.3 (1.3-7.8)	0.57	18.8 (16.5-40.6)	31.3 (17.4-42.0)	0.16	<0.0001	<0.0001
altitude (1<3000m,2:3000-4000m,3≥4000m)			0.38			0.53	0.06	0.45
1	3	2		2	1			
2	27	52		29	24			
3	0	1		6	2			
Profession (1:teacher,2:student,3:army,4: civil servant,5:worker, 6:farmer)			0.17			0.005	<0.0001	<0.0001
1	6	7		5	4			
2	0	0		23	7			
3	11	37		0	2			
4	2	4		4	4			
5	7	5		0	7			
6	4	2		5	3			
diet (1:meat,2:vegetable,3:balanced)			0.41			0.08	0.0001	0.016
1	4	10		18	12			
2	4	3		11	3			
3	22	42		8	12			
Alcohol(1:never,2:seldom,3:often)			0.73			0.63	0.015	0.14
1	13	19		5	6			
2	16	34		27	17			
3	1	2		5	4			
water intake(1<1000ml,2:1000-2000ml;3≥2000m)			0.45			0.40	0.09	0.13
1	10	20		5	5			
2	13	28		25	20			
3	7	7		7	2			
exercise (1:never,2:seldom,3:often)			0.16			0.34	0.003	0.06
1	2	1		1	3			
2	11	31		29	18			
3	17	23		7	6			
Gout (n,%)	0	5	0.16	2	2	0.74	0.20	0.80
Hypertension (n,%)	0	1	0.46	6	4	0.88	0.021	0.021
Pulmonary arterial hypertension(n,%)	0	0		0	0			
Biliary/renal calculus(n,%)	0	1	0.46	1	0	0.39	0.36	0.48
Gastropathy (n,%)	5	3	0.09	8	2	0.12	0.61	0.73
Hepatitis (n,%)	0	1	0.46	1	0	0.39	0.36	0.48
Tuberculosis (n,%)	0	1	0.46	0	2	0.09	-	0.21

Intriguingly, in the normal UA populations of Group A, the Tibetans had heavier BW in contrast with shorter height than the Hans, leading to higher BMI than the Hans (**Table 2**). The Tibetans had different professional compositions and dietary habits, but more alcohol consumption and were more likely to develop hypertension in both normal and high UA populations, and less exercise than Hans in normal UA populations.

In Group B, the Hans (n=70) were also younger than Tibetans (n=156) [33 (24-47) *vs.* 42 (33-52) (year), *P*=0.004]. The incidence of hyperuricemia was 44.3% in the Hans and 32.7% in the Tibetan.

In Group C, the Hans (n=46) were slightly older than native Tibetans (n=65) (*P*=0.019). The Hans had a higher height and heavier BW than the Tibetans, resulting in an identical BMI. The incidence of hyperuricemia was 40.0% in the Hans and 18.2% in the Tibetan. Both populations had similar blood pressure and heart rates (**Table 3**). The Tibetans differed from the Hans in an extremely long Tibetan dwelling time (year) [19.0 (18.0-20.0) *vs.* 2.0 (1.0-4.0), *P*<0.001], more meat in diet and water intake, more tap water over purified water, and less exercise (all *P*<0.001). Nonetheless, Tibetans had fewer signs of acute and chronic altitude sickness.

**Table 3:** Comparison of the biochemistry between the Hans and the Tibetans in Naqu, Chinese Tibetan region (Group C).

Variate	Age (y)	Dwelling time (y)	Height (cm)	Body weight (kg)	BMI	WBC (10^9^/L)	Eosinophil (10^9^/L)	Lymphocyte (10^9^/L)	Neutrophil (10^9^/L)	Platelet (10^9^/L)	MPV (fL)	P-LCR%
Tibetan, median (IQR)	19 (18-20)	19 (18-20)	164 (161-168)	56.0 (50.5-22.0)	20 (19-22)	7.65 (5.8-9.5)	0.5 (0.4-0.7)	1.60 (1.38-2.00)	5.5 (3.8-7.0)	239.5 (184.3-259.8)	9.6 (8.8-10.2)	0.22 (0.17-0.26)
Hans, median (IQR)	20 (19-22)	2 (1-4)	170 (168.3-175)	60.5 (55.0-67.0)	21 (19-22)	8.15 (5.95-10.8)	0.6 (0.5-0.8)	1.65 (1.48-2.10)	5.7 (3.8-7.7)	215.5 (166.8-256.8)	9.8 (9.3-11.3)	0.24 (0.19-0.35)
*P* value (*t* test)	0.019	<0.001	<0.001	<0.001	0.40	0.57	0.55	0.59	0.65	0.32	0.036	0.038
Variate	RBC (10^12^/L)	SPO2	Hematocrit (%)	Hemoglobin (g/L)	MCV (fL)	MCH (pg)	MCHC (g/L)	RDW-SD (fL)	RDW-CV (%)	RDW %	Uric acid (μmol/L)	Glucose (mmol/L)
Tibetan	5.57 (0.7)	86 (84-88)	155 (139-200)	0.49 (0.47-0.50)	89.5 (87.3-91.5)	32.5 (1.5)	357 (352-364)	43.2 (3.5)	0.140 (0.134-0.143)	11.2 (10.2-12.6)	373 (362-382)	3.70 (0.10)
Hans	6.0 (0.6)	88 (83-90)	201 (182.5-220.5)	0.54 (0.50-0.58)	89.0 (85.9-92.1)	32.9 (2.6)	369 (352-378)	43.2 (4.1)	0.138 (0.131-0.143)	12.1 (10.7-14.2)	400 (361-489)	3.31 (0.44)
*P* value (*t* test)	0.010	0.71	0.018	0.004	0.73	0.38	0.76	0.94	0.93	0.40	0.37	0.22
Variate	Total protein (g/L)	Albumin (g/L)	globulin (g/L)	TBIL (μmol/L)	DBIL (μmol/L)	IBIL (μmol/L)	ALT (U/L)	ALP (U/L)	GGT (U/L)	Urea (mmol/L)	Creatinine (μmol/L)	
Tibetan	82.3 (3.8)	48.8 (47.6-49.9)	33.0 (29.6-35.5)	11.1 (9.4-15.4)	4.4	6.5 (5.4-8.3)	23 (17-30)	102 (79-141)	23 (21-26)	8.85 (1.95)	94.0 (14.0)	
Hans	80.2 (4.7)	49.2 (47.6-50.8)	30.8 (28.9-33.8)	14.8 (11.0-22.2)	4.95	9.8 (7.1-14.8)	24.5 (20.0-40.0)	93 (73-119)	19 (17-24)	8.21 (1.17)	93.3 (10.2)	
*P* value (*t* test)	0.18	0.20	0.004	0.003	0.052	<0.001	0.20	0.27	0.52	0.36	0.16	

Data are expressed as median (IQR) or median (SD). SBP, Systolic blood pressure; DBP, diastolic blood pressure; BMI, body mass index; RDW, red cell distribution width; MPV, mean platelet volume; BIL, bilirubin; WBC, white blood cell; RBC, red blood cell; SPO2, blood oxygen saturation; MCV, mean corpuscular volume; MCH, mean corpuscular hemoglobin; MCHC, mean corpuscular hemoglobin concentration; RDW, red distribution width; SD, standard deviation; CV, coefficient of variation; MPV, mean platelet volume; P-LCR, platelet-larger cell ratio; ALT, alanine aminotransferase; AST, aspartate aminotransferase; GGT, γ-glutamyl transferase; TBIL, total bilirubin; DBIL, direct bilirubin.

### Distinct risk factors associated with serum UA between the Hans and the Tibetans

Consistent with previous reports (1, 29), almost all risk factors associated with MS were significantly associated with UA levels in the Han population, including age, altitude, BMI, aminotransaminase, the product of heme catabolism (bilirubin), cholesterol transporter (HDL), a biomarker for glomerular filtration rate (cystatin C), glucose, and insulin levels (**Table 4**). Meanwhile, factors that were not significantly associated with serum UA levels in Hans were lipid metabolism and transportation (total TRIG, TCHOL, and LDL-C3), renal function (creatinine and urea), metabolism of extracellular nucleotides (ALP), and glycolysis (LDH) (**Table 4**). Unexpectedly, none of the above factors contributed to UA levels in Tibetans, suggesting a distinct mechanism underlying hyperuricemia in native Tibetans.

**Table 4:** Multivariate line regression analysis of the risk factors associated with serum uric acid levels between Hans and Tibetans in Tibet, China (Group A).

Ethnic		Chinese-Tibetans			Chinese-Hans	
Variable	OR	95% CI	P value	OR	95% CI	P value
Age	1.29	-10.22 to 2.659	0.22	4.78	-8.316 to -3.333	<0.001
Altitude	0.15	-91.17 to 79.90	0.89	2.54	16.02 to 148.2	0.017
BMI	0.28	-8.118 to 6.295	0.79	2.84	1.912 to 11.79	0.008
Albumin	0.80	-17.93 to 8.383	0.44	2.26	0.6394 to 13.01	0.032
ALT	0.14	-3.176 to 2.794	0.89	2.68	-6.590 to -0.8812	0.012
AST	0.23	-6.670 to 8.198	0.83	2.63	1.446 to 11.52	0.013
GGT	0.58	-2.516 to 1.460	0.57	2.61	0.5106 to 4.235	0.014
ALP	1.21	-0.7793 to 0.2275	0.25	0.91	-1.145 to 0.4411	0.37
TBIL	1.01	-16.19 to 43.56	0.34	4.34	-26.58 to -9.549	<0.001
DBIL	0.71	-104.9 to 53.82	0.49	4.35	28.98 to 80.32	<0.001
TRIG	0.21	-135.4 to 164.4	0.83	0.56	-27.31 to 47.94	0.58
CHOL	0.65	-462.1 to 251.2	0.53	0.06	-113.4 to 107.5	0.96
HDL-CH	0.54	-393.1 to 238.7	0.60	2.37	-321.4 to -23.80	0.025
LDL-CH	0.53	-288.0 to 469.4	0.61	0.40	-91.44 to 136.0	0.69
Creatinine	0.44	-4.761 to 7.131	0.67	1.40	-0.4563 to 2.441	0.17
Cystatin C	1.28	-156.6 to 589.1	0.23	4.80	241.2 to 599.7	<0.001
UREA	0.05	-33.79 to 35.23	0.96	0.74	-13.81 to 6.488	0.47
Glucose	0.30	-44.12 to 33.40	0.77	2.26	3.002 to 59.01	0.031
LDH	0.54	-1.273 to 0.7681	0.60	0.33	-0.3414 to 0.4751	0.74
Insulin	1.09	-2.001 to 5.896	0.30	3.14	-3.128 to -0.6621	0.004

OR, odds ratio; CI, confidence interval; BMI, body mass index; ALT, alanine aminotransferase; AST, aspartate aminotransferase; GGT, γ-glutamyl transferase; ALP, alkaline phosphatase; TBIL, total bilirubin; DBIL, direct bilirubin; TRIG, triglyceride; TCHOL, total cholesterol; HDL-CH, high-density lipoprotein cholesterol; LDL-CH, low-density lipoprotein cholesterol; LDH, lactate dehydrogenase.

### The Tibetans showed heightened insulin resistance and glycolysis compared to the Hans

**Figure 1:**
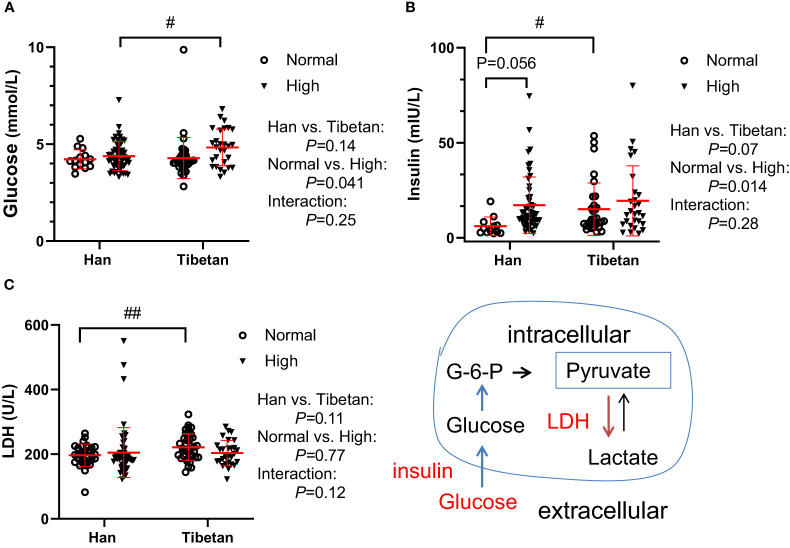
Comparison of glucose metabolism between Hans and Tibetans with normal or high uric acid (UA) levels in Group A. LDH, lactate dehydrogenase. #*P*<0.05, ##*P*<0.01 Tibetans *vs.* Hans.

The normality of biochemical indicators in the three groups was tested (**Supplementary 1**). We then investigated glycolysis, which may allow ATP to be rapidly generated in hypoxic cells. In Group A, although the glucose levels were similar between the Hans and Tibetans in the normal UA populations, they were increased by high UA exclusively in the Tibetans. Conversely, both fasting insulin (*P*<0.05) and LDH (*P*<0.01) levels were higher in Tibetans than in Hans in normal UA populations (**Figure 1**). These results indicate an increased tendency of insulin resistance and subsequent glycolysis in Tibetans relative to Hans.

At higher altitudes, as in Group C, the glucose level was further decreased in the young adults of Hans [3.3 (3.1-3.6) *vs*. 4.2 (3.9-4.5) mmol/L, *P*<0.001] but not in the Tibetans [3.70 (3.6-3.8) *vs.* 4.1 (3.9-4.4) mmol/L, *P*=0.45] compared with the respective ethnic groups in Group A with normal UA levels. However, the glucose levels were comparable between the two ethnicities in Group C) (**Table 3**).

### The Tibetans had reduced lipid and biliary metabolism compared with the Hans

In both groups of our populations, the level of total cholesterol was 3.21-4.48mmol/L (124-173mg/dl), LDL cholesterol was between 1.85-2.51mmol/L (77-97mg/dl), both were much lower than the total cholesterol (224.0 ± 42.9 mg/dl) and LDL cholesterol (145.3 ± 39.3 mg/dl) from an Italian population, respectively. However, the level of HDL-cholesterol was between 1.00-1.18mmol/L (37-44 mg/dl) and TG was between 0.86-2.24 mmol/L (55-143mg/dl), similar to those from the same Italian population with HDL (55.6 ± 15.6 mg/dl) and TG [96 (69-137) mg/dl], respectively (30).

**Figure 2:**
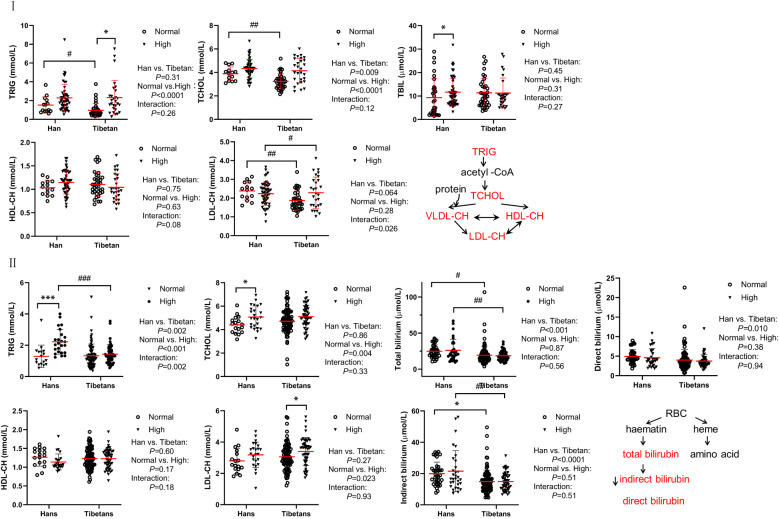
Comparison of lipid metabolism and transporters between Hans and Tibetans with normal or high uric acid (UA) levels in Group A (**I**) and Group B (**II**) I: Tibetans showed lower triglyceride (TRIG), total cholesterol (TCHOL), total bilirubin (TBIL), and low-density lipoprotein-cholesterol (LDL-CH) levels than Hans in normal UA populations. Total bilirubin (TBIL) and high-density lipoprotein cholesterol (HDL-CH) were identical between the two populations. II: Tibetans showed lower levels of TRIG, TBIL, and direct and indirect bilirubin than Hans in both normal and high UA settings. TRIG, TCHOL and LDL-CH were increased by hyperuricemia in both populations. TCHOL, HDL-CH, and LDL-CH were similar between the Hans and the Tibetans. *P<0.05, ***P<0.001, normal UA *vs.* high UA; #*P*<0.05, ##*P*<0.01, ###*P*<0.001, Tibetans *vs.* Hans.

Surprisingly, in Group A (**Figure 2I**), the lipid parameters, including TRIG, TCHOL, and LDL-CH, were significantly lower in Tibetans than in Hans, even in the normal UA setting. Both TRIG and TCHOL levels were significantly increased in the high UA population. TBIL and HDL levels were identical between the Hans and Tibetans, except that TBIL was only increased by high UA levels in the Hans. In Group B, at higher altitudes, it was noteworthy that TRIG, total, direct, and indirect bilirubin levels were significantly higher in the Hans than in the Tibetans (**Figure 2II**). Likewise, in Group C from even higher altitudes of Naqu, Tibetans showed consistently lower levels of total, direct, and indirect bilirubin than Hans (**Table 3**). Bilirubin is a breakdown product of heme released during RBCs lysis (31). In normal UA populations, the relatively lower levels of bilirubin in Tibetans relative to Hans indicated reduced RBCs hemolysis in Tibetans (**Table 3**; **Figure 2II**).

### Distinct biochemistry biomarkers for protein metabolism between Hans and Tibetans

**Figure 3:**
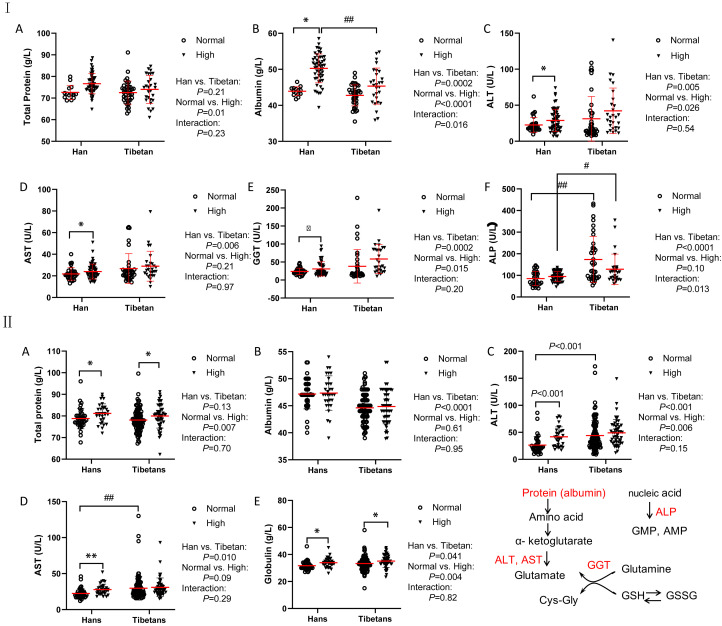
Comparisons of the biomarkers for protein metabolism between the Hans and Tibetans with normal or high uric acid (UA) levels in Group A (**I**) and Group B (**II**). The Tibetans exhibited lower levels of albumin but higher levels of aminotransferase than the Hans in both normal and high UA settings in both Group A and Group (B) *P<0.05, **P<0.01, normal UA *vs*. high UA; #*P*<0.05, ##*P*<0.01, Tibetans *vs.* Hans.

In Groups A and B, although the total protein levels were similar between the Han and Tibetan populations, they were increased by high UA levels in both populations (**Figures 3I, II**). Conversely, the albumin levels were elevated by high UA in both ethnic groups in Group A (**Figure 3I**), but were still lower in the Tibetans than in the Hans, regardless of UA level in both Groups A and B (**Figures 3I, II**). Biochemistry analysis revealed higher levels of all aminotransaminase and ALP in Tibetans than in Hans in both Group A (ALT, AST, GGT, and ALP) and Group B (ALT and AST). Intriguingly, the levels of aminotransaminase were elevated by high UA exclusively in the Hans (**Figures 3IC–E**, **3IIC, D**). In Group C, Tibetans had higher total protein and globulin levels than Hans, but identical levels of aminotransaminases (**Table 3**).

### The Tibetans had less severe kidney injury than the Hans

**Figure 4:**
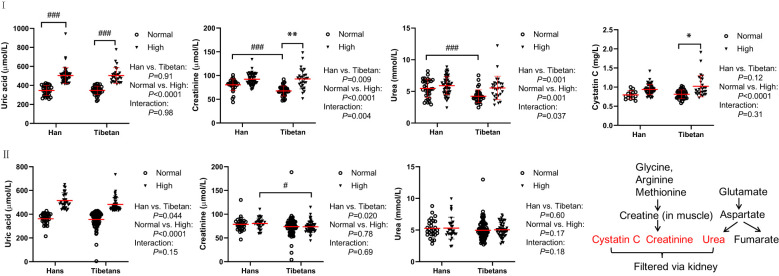
Comparisons of the biomarkers for kidney and heart injury between Hans and Tibetans with normal or high uric acid (UA) levels in Group A (**I**) and Group B (**II**). *P<0.05, **P<0.01, normal UA *vs.* high UA; #*P*<0.05, ###*P*<0.001, Tibetans *vs.* Hans.

In Group A, UA levels were comparable between the Hans and Tibetans in both the normal and high UA populations (**Figure 4IA**). Tibetans had lower levels of creatinine and urea than Hans, but cystatin C levels were similar to those of Hans (**Figures 4IB–D**). All the levels of the above biomarkers were greatly increased by high UA levels in Tibetans. However, the ethnic differences, especially the differences between the normal and high UA groups, were less significant in Group B than in Group A (**Figure 4II**). Although the levels of urea and creatinine UA were higher in high UA than in normal UA populations in Groups A and B, they were identical between Tibetans and Hans in young adults in Group C (**Table 3**). As the levels of biomarkers for kidney injury increased with altitude in the Han population, the difference between the two ethnic populations seemed to be attenuated with the increase in altitude.

Creatine kinase (CK) and its MB isoenzyme (CK-MB) are the most commonly used serological biomarkers for the diagnosis of myocardial infarction. In Group A, CK did not differ between Tibetans and Hans regardless of the UA level (**Figure S2A**). CK-MB was increased by high UA levels exclusively in Tibetans (**Figure S2B**). N-terminal fragment B-type natriuretic peptide (NT-pro-BNP) is frequently used for the diagnosis of congestive heart failure. NT-proBNP levels are affected by age or the presence of one or several comorbidities, such as chronic renal failure, type 2 diabetes, and acute coronary syndrome (32). In the Hans of Group A, we detected decreased NT-pro-BNP levels in the high UA group compared to the normal UA group (**Figure S2C**), its significance remains unclear.

### The Tibetans had lower serum levels of thyroxin than the Hans

**Figure 5:**
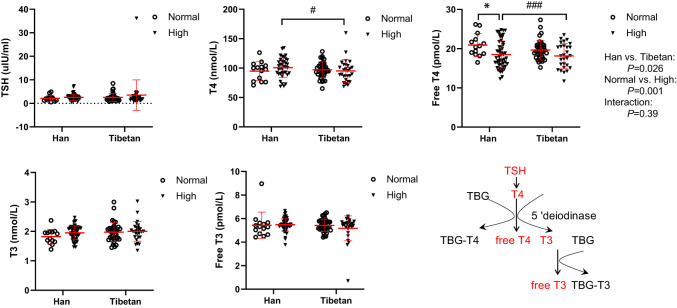
Comparisons of serum thyroxin levels between Hans and Tibetans with normal or high uric acid (UA) levels in Group A. *P<0.05, normal UA *vs.* high UA; #*P*<0.05, ###*P*<0.001, Tibetans *vs.* Hans. TBG, thyroxin-binding globulin.

Regardless of the UA level, thyroid-stimulating hormone (TSH), triiodothyronine (T3), and free T3 levels were not altered between the Hans and Tibetans in Group A (**Figure 5**). Although L-thyroxin (T4) levels were similar between the Han and Tibetan populations in normal UA populations, they were lower in Tibetans than in Hans in high UA populations. Similarly, serum free T4 levels were lower in Tibetans than in Hans in high-UA populations. It appears that hyperuricemia exempts Tibetans from T4 synthesis and the release of free T4.

### The Tibetans had lower serum levels of electrolytes than the Hans

**Figure 6:**
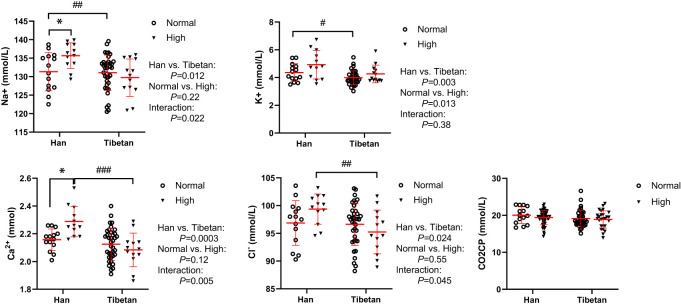
Comparisons of serum sodium, potassium, calcium and chlorine concentrations between Tibetans and Hans with normal or high uric acid (UA) levels in Group A. *P<0.05, normal UA *vs.* high UA; ^#^ *P*<0.05, ^##^ *P*<0.01,^###^ *P*<0.001, Tibetans *vs.* Hans. CO2CP: carbon dioxide combining power.

In Group A, all serum electrolyte levels were much lower in Tibetans than in Hans in the normal and high UA populations (**Figure 6**). Interestingly, the electrolyte levels tended to be elevated by high UA levels exclusively in the Hans rather than in the Tibetans. This implies that the proximal convoluted tubule, which is responsible for electrolyte absorption, is more resilient to the dangerous effects of hyperuricemia in Tibetans than it is in Hans.

### Similar circulating hemolytic counts between the Tibetans and the Hans

**Figure 7:**
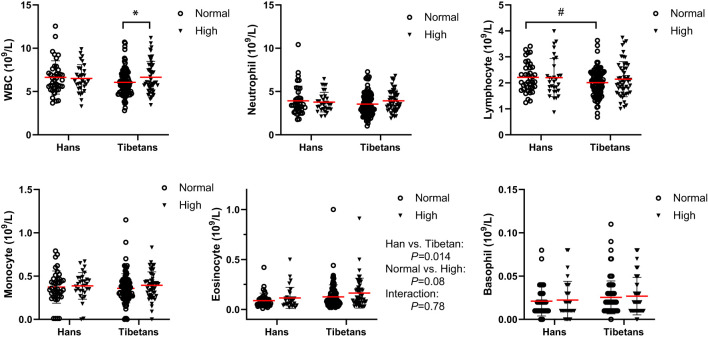
Comparisons of the blood cell counts between Tibetans and Hans with normal or high uric acid (UA) levels in Group (B) *P<0.05, normal UA *vs.* high UA; ^#^ *P*<0.05.Tibetans *vs.* Hans.

In Group B, Tibetans had similar counts of blood cells as Hans, except that they had fewer lymphocyte counts but more eosinophil counts than Hans (**Figure 7**). Tibetans with high UA had increased white blood cell (WBC) counts in comparison with those in the normal UA group. Likewise, in Group C of young adults, Tibetans were not distinguished from Hans in blood cell counts (**Table 3**). The normality of blood leukocyte parameters in Group C was also tested (**Supplementary 1**).

### The Tibetans exhibited less erythropoiesis than the Hans under hyperuricemia

**Figure 8:**
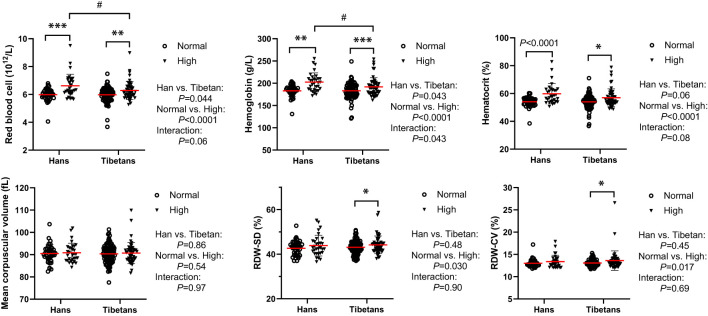
Tibetans showed lower levels of erythropoiesis than Hans with normal or high uric acid (UA) levels in Group (B) *P<0.05, **P<0.01, ***P<0.001, normal UA *vs.* high UA; ^#^ *P*<0.05, Tibetans *vs.* Hans.

In Group B, although both RBC number and [Hb] were beyond the reference ranges (male: RBC 4.3-5.9×10^12^/L, [Hb] 137179 g/L), they were indistinguishable between the two populations under normal UA conditions. Both were differentially increased between Tibetans and Hans under high UA conditions, resulting in higher levels in Hans than in Tibetans (**Figure 8**). RBC volume distribution width (RDW) is a conventional biomarker of erythrocyte volume variability and an indicator of homeostasis (33). The RDW standard deviation (SD) and coefficient of variation (CV) were elevated by high UA only in Tibetans (**Figure 8**).

In Group C, the Hans rather than the Tibetans developed polycythemia with extremely high [Hb] and hematocrit values beyond the normal ranges. In comparison, Tibetans had lower [Hb] values, reduced RDW%, smaller mean platelet volume (MPV) and lower platelet-larger cell ratio (P-LCR) % than Hans (**Table 3**), indicating greater resilience to severe hypoxia at higher altitudes in Tibet. This implies that severe hypoxia distinguishes the two ethnic populations in the RBC profile, similar to the high UA level in RBC in **Figure 8**.

Interestingly, in Group B, although the UA levels correlated with RDW-SD and RDW-CV significantly in Tibetans (**Table S1**), the RBCs profiles were not independently associated with UA levels in either Tibetans or Hans (**Table 5**). Similar to that in Group A (**Table 4**), only the glucose level was associated with the UA level in Hans other than Tibetans (**Table 5**).

**Table 5:** Multivariate line regression analysis of the blood parameters associated with serum uric acid levels between Hans and Tibetans in Tibet, China (Group B).

Variable	Hans			Tibetan		
	OR	95% CI	P value	OR	95% CI	P value
age	1.303	-5.461 to 1.183	0.20	0.728	-2.062 to 0.9534	0.47
RBC	0.066	-452.6 to 483.0	0.95	1.068	-261.3 to 873.8	0.29
HGB	0.478	-44.73 to 27.62	0.64	0.310	-33.32 to 24.30	0.76
HCT	0.722	-61.37 to 129.4	0.47	0.244	-122.3 to 95.48	0.81
MCV	0.571	-291.2 to 163.0	0.57	0.043	-0.02467 to 0.02577	0.97
MCH	0.629	-451.1 to 857.9	0.53	1.033	-49.98 to 159.1	0.30
MCHC	0.435	-69.79 to 45.07	0.67	0.374	-22.17 to 15.12	0.71
RDW-SD	0.546	-19.99 to 11.49	0.59	0.100	-34.53 to 31.20	0.92
RDW-CV	0.257	-2.185 to 1.693	0.80	0.280	-93.71 to 124.6	0.78
Glucose	2.248	3.945 to 74.65	0.030	1.491	-3.600 to 0.5060	0.14

OR, odds ratio; CI, confidence interval; RBC, red blood cell; HGB, hemoglobin; HCT, hematocrit; MCV, mean corpuscular volume; MCH, mean corpuscular hemoglobin; MCHC, mean corpuscular hemoglobin concentration; RDW, red cell distribution width; SD, standard deviation; RDW-CV, RDW-coefficient of variation.

## Discussion

Although the prevalence of hyperuricemia is much higher in Tibet than in other places in China, immigrant Hans have accumulated a high incidence of hyperuricemia in Tibet. When we compared the incidence of hyperuricemia between native Tibetans and immigrant Hans at the three altitudes in the Tibetan region, we found a similarly high prevalence of hyperuricemia between the two ethnicities but with distinct biochemical mechanisms. Hyperuricemia is closely associated with obesity and metabolic disturbances such as insulin resistance, dyslipidemia, hypertension, and kidney disease in lowlanders (34) and highlanders in Tibet (1) and Peru (35).

In our study, the high prevalence of hyperuricemia in high-altitude-adapted native Tibetans reached at the similar level as the patients with Acute Coronary Syndrome, e.g., acute Heart Failure (35.8%) (36), which could not be explained simply by traditional metabolic disturbances. First, the factors that were significantly associated with UA levels in the Han population were not applied to Tibetans. Second, Tibetans had higher serum insulin and LDH levels, indicating heightened anaerobic metabolism compared to Hans. Third, Tibetans had increased aminotransferase and ALP activities, suggesting enhanced protein and nucleic acid turnover compared with Hans. Fourth, Tibetans had extremely low serum levels of TRIG, TCHOL, and LDL-CH, implying a lower degree of lipometabolism. Finally, Tibetans had better hypoxic adaptation with a lower degree of polycythemia than Hans. The above biochemical discrepancy between the two populations may be distinctively associated with hyperuricemia between Tibetans and Hans.

### Purine metabolism in hypoxia

UA is primarily produced in the liver as the end product of exogenous and endogenous purine metabolism, covering the catabolism, *de novo* synthesis, and salvage pathways (37). The intake of fructose or a purine-rich diet, ATP depletion induced by ischemia, and degradation of RNA and DNA can activate the purine metabolism pathway (38).

The high prevalence of hyperuricemia in Tibetans may be attributed to the activation of the purine metabolism pathways. In hypoxia, ATP production is hindered by a lack of oxygen, which accelerates the breakdown of adenosine monophosphate (AMP) to maintain energy levels (26). Under normal conditions, the majority of hypoxanthine is reutilized through the salvage pathway. Under hypoxia, the rate of salvage and degradation decreases because of energy deficiency, which results in hypoxanthine accumulation (39). Therefore, we speculated that metabolic adaptation to hypoxia may contribute to hyperuricemia in Tibetans.

Serum alkaline phosphatase (ALP) is a traditional indirect marker of cholestasis. ATP, adenosine diphosphate (ADP), and AMP can be metabolized to adenosine by two different enzyme systems. Ecto-5-nucleotidase (CD73) converts AMP to adenosine (40) and tissue-nonspecific ALP proteins catabolize nucleotides in a nonspecific manner (41). Soluble adenosine deaminase catabolizes adenosine to inosine. Therefore ALP is involved in the regulation of purinergic signaling by participating in the degradation of extracellular nucleotides (42). It has been reported that plasma adenosine concentration and soluble CD73 activity rapidly increase at high altitude (43). Similarly, serum ALP levels increase with serum UA levels in patients with peripheral arterial disease (44). In line with this report, we demonstrated that ALP levels were greatly increased in Tibetans relative to Hans in both the normal and high UA groups. Higher ALP levels may be involved in purine metabolism by mediating nucleotide degradation in Tibetans.

### Metabolic adaptation by promoting glycolytic capacity

At the cellular level, the response to hypoxia results in the promotion of glycolytic capacity, increased glycolytic flux, and lactate efflux in cells (45). LDH is a ubiquitously expressed enzyme that reversibly catalyzes the reduction of pyruvate to L-lactate in the Cori cycle (46). Consistent with elevated muscle LDH activity in highlanders in Sherpas, Nepal (18), native Tibetans had higher LDH levels than Hans in normal UA populations (**Figure 2I**). This result indicated that Tibetans obtained enhanced glycolysis and gluconeogenesis compared with Hans to maintain adequate ATP levels in a hypoxic environment.

Accordingly, compared with Hans, Tibetans had higher insulin levels in the normal UA population and higher fasting glucose levels in the high UA population in our study (**Figure 1**). This finding is interesting because the prevalence of diabetes was significantly lower in Tibetans than in Hans in China (47), and people dwelling at high altitudes had a lower diabetes prevalence than those living at low altitudes (48). However, it was also found that fasting glucose level was significantly higher in the high UA group than in the normal UA group on the Tibetan Plateau (1), and Tibetan highlanders may be vulnerable to glucose intolerance in both China and India (49). Our results suggest that hyperuricemia may modify this protection in subjects at high altitudes, thus increasing the risk of glucose intolerance. Accordingly, the free T4 level was reduced in Tibetans compared with Hans in high UA populations, indicating a relatively lower metabolic rate (glycolytic flux) in this population of Tibetans.

Accumulating evidence suggests that hyperuricemia is associated with impaired glucose metabolism (50) and insulin resistance (51). Hyperinsulinemia can lead to hyperuricemia, but not vice versa (52). Reducing the glycemic quality of carbohydrates over five weeks could reduce UA levels in American subjects (53). Recently, an inverted U-shaped association was observed between major glycemic indices and UA levels in the Chinese population, in which UA levels were elevated with increasing glycemic indices before the inflection points and then decreased with further increases in glycemic indices (54).

In hypoxia-tolerant systems, a shift away from fatty acid oxidation toward a more oxygen-efficient hypometabolic pathway with the downregulation of ATP demand is a common strategy (45). In rats, exposure to hypoxia resulted in the downregulation of fatty acid oxidation and increased pyruvate oxidation (55). Here, we provided corroborative evidence that Tibetans had consistently lower levels of lipometabolism than Hans (**Figure 2**), indicating better hypoxia tolerance in Tibetans.

### Amino acid metabolism

In the catabolic state of insulin resistance, AST and ALT are responsible for transferring amino acid groups to produce essential intermediate products in the gluconeogenesis pathway. ALT, frequently referred to as glutamic pyruvate transaminase, catalyzes the reversible transamination of alanine and α-ketoglutarate into glutamate and pyruvate (46). Therefore, alanine is a major gluconeogenic precursor. Similar to and often parallel with lactate in the Cori cycle, pyruvate is transformed into alanine by transamination in the muscle, and then alanine is deaminated back to pyruvate in the liver. Therefore, disorders of glucose metabolism are strongly related to liver enzyme abnormalities; for example, AST/ALT levels are inversely correlated with the occurrence of type 2 diabetes (56).

Saliva ALT and glutamic oxaloacetic transaminase levels increase after hypobaric hypoxia in healthy military aircrews (57). Hypoxia-reoxygenation results in the release of LDH, AST, ALT, and XO in the liver of rats (58). Markers of hypoxia correlated significantly with AST and ALT levels in patients with obstructive sleep apnea (59). Specifically, ALT and AST levels were significantly higher in the high UA group than in the normal UA population on the Tibetan Plateau (1). Likewise, regardless of UA levels, we found elevated levels of ALT and AST in Tibetans relative to Hans, in contrast with lower levels of albumin (**Figures 3IB**, **3IIB**) and urea (**Figure 4I**) in Groups A and B, instead of in Group C. The discrepancy between Groups A, B and C might have contributed to the higher BMI in Tibetans than in Hans in the former two groups and similar BMI between Tibetans and Hans in Group C. These findings indicate that enhanced amino acid utilization and transamination may underlie hyperuricemia in Tibetans.

Glutathione (GSH) is the principal intracellular antioxidant buffer against oxidative stress in the form of reduced GSH and oxidized GSH (GSSG) (19). A favorable reduced/oxidized GSH ratio (GSH/GSSG) is required for cytosolic antioxidant defenses. In short-term exposure to hypoxia, GSH/GSSG was only increased in the muscle of lowlanders but not in highland Sherpa, indicating superior redox homeostasis in highlanders (18). Under hypoxia, the ratio of GSH/GSSG was increased in RBCs (60). Recently, it was found that RBC rely on glutamine to fuel GSH synthesis and pyruvate transamination during hemorrhagic shock (61). GGT is a cell surface enzyme that hydrolyzes the γ-glutamyl bond of extracellular reduced and oxidized GSH into glutamate, cysteine (Cys), and glycine (Gly) (62). In line with the heightened GGT level at high altitudes (35), we found that GGT levels were much higher in Tibetans than in Hans. Moreover, higher GGT levels were reported in the group with impaired fasting glucose than in those with normal fasting glucose in the Chinese population (63), and GGT level was increased to a greater extent by high UA in Hans than in Tibetans (**Figure 3I**), indicating increased cleavage of GSH in Hans other than Tibetans. Our findings recapitulated the assumption of superior redox homeostasis in the highlander, for example, the Tibetans in our study (18).

### PPP in RBCs

The hematological response to hypoxia is characterized by erythropoiesis, which leads to an increased [Hb] value that increases the oxygen-carrying capacity. It has been reported that native Tibetans have lower [Hb] than Han immigrants (64, 65), which is associated with the positively selected haplotypes of the egl-9 family hypoxia-inducible factor 1 and *PPARA* (66). In Chinese Tibetan immigrants, [Hb] was a positive risk factor for high UA level (5). Accordingly, we found that native Tibetans had lower [Hb] than immigrant Hans under both normal and high UA conditions (**Figure 7**; **Table 3**), suggesting a distinctive ethnic difference in the hematological response to altitude.

Recently, it was speculated that the purinergic system may be involved in metabolic adaptations of RBCs to hypoxia. PPP generates ribose sugars for nucleotide synthesis (19). Hypoxia not only promotes glycolysis, but also deregulates PPP and depresses purine catabolism, glutathione homeostasis, and arginine/nitric oxide metabolism in RBCs (60). Likewise, hypoxia can divert glucose to PPP in the muscle to mitigate the effects of adenosine degradation (19). Consequently, the accumulation of AMP, adenosine, and the PPP product ribose 5-phosphate (ribose-5-P) may activate *de novo* synthesis of purines (67).

In humans, exposure to hypoxia immediately increases RBC glycolysis while shutting down PPP (60). The transient increase in ATP levels during the early response to hypoxia resulted in the accumulation of AMP, adenosine, and the PPP product ribose-5-P in RBCs, proportional to the duration of high-altitude exposure (60). Therefore, severe polycythemia, which is a sign of poor hypoxic adaptation, may be associated with increased UA levels in the Han population (**Figure 8**; **Table 3**). In Group B, although the RBC parameters correlated well with UA levels in the two ethnic populations (**Table S1**), none was independently associated with UA levels in either group (**Table 5**). However, blood glucose levels were consistently associated with UA levels only in Hans (**Tables 4**, **5**). People with polycythemia seem to be particularly vulnerable to glucose intolerance (68), suggesting poorer adaptation to hypoxia in people with polycythemia than in those without. It should be explored whether severe polycythemia is possibly associated with a high incidence of hyperuricemia in the Hans, as in Group C (**Table 3**).

### Kidney function

One of the mechanisms underlying hyperuricemia is insulin resistance, which causes a significant decrease in the urinary excretion of UA, sodium, and potassium (69, 70). In healthy individuals, most glucose filtered at the glomerulus is reabsorbed into the epithelial cells from the glomerular filtrate *via* the sodium-glucose cotransporter (SGLT) in the kidney. Glucose then passes into the interstitial fluid and peritubular capillary *via* the glucose transporter maintained by Na+/K+ ATPase in the proximal tubule (71). In our study, in normal UA populations, the higher insulin level in Tibetans relative to Hans indicated possibly worsened insulin resistance in Tibetans. In light of these studies, it is possible that the diabetogenic state in Tibetans would prevent glucose and sodium reabsorption *via* SGLT, resulting in lower serum sodium concentrations. The disparity in serum electrocytes between the two ethnicities was exacerbated by the high UA levels. These results suggest that more severe insulin resistance in Tibetans may be associated with reduced sodium and potassium reabsorption (**Figure 6**).

Overall, Tibetans had increased glucose metabolism at the expense of lower fatty acid oxidation under anoxia, resulting in enhanced glycolysis and gluconeogenesis, triggering glucose conversion to ribose-5-P, an essential component of nucleotide synthesis *via* PPP (19). Increased glycolysis also promotes the transamination of amino acids, resulting in enhanced gluconeogenesis. Consequently, the higher ALP levels triggered by hypoxia may mediate the degradation of nucleotides, thus increasing the production of purine nucleotides. On the other hand, the immediate shutdown of PPP in RBCs to high altitudes may activate the purinergic system, which may be associated with hyperuricemia in the Hans, who exerted poorer hypoxic adaptation than the Tibetans.

### Study strengths and limitations

These findings are important for the management of metabolic adaptations in hypoxia-related diseases in critical care settings. Our study provides thorough descriptions and comparisons of the biochemical differences between native Tibetans and immigrant Hans in three relatively young populations simultaneously at different altitudes in Tibet, China. Based on these findings, we propose a distinctive etiology underlying the ethnic disparity in hyperuricemia in the Tibetan Plateau. Moreover, we provide corroborative evidence for previous high-altitude adaptation and highlight the complexity of hypoxia-response pathways in humans. Even so, the study on the influence of the environment in Tibetan areas may not offset the influence of genetic background. Future study on the interaction between environment (disease) adaption and ethnicity would provide more information for managing the critical illness.

Although we recruited both sexes in the beginning, for the limitation of the page space, we only present the data from the male population here. The results including the female population would reflect the biochemistry of hyperuricemia at the overall level of population. Because of the religious faith, it is not easy to collect the blood samples from a larger crowd in Tibetan areas, the limitation of insufficient effect due to the small sample size should be considered. In the multiple line regression analysis, other demographic factors such as the economic status, education, and profession may also be the potential confounding factors but were not included in this study. Another limitation was that routine blood tests, including white blood cell count, red RBCs, and [Hb], were not conducted in Group A.

## Conclusions

The risk factors associated with MS for hyperuricemia in immigrant Han individuals did not apply to native Tibetans on the Chinese Tibetan Plateau. However, the higher ALP activity in Tibetans than in Hans may be involved in purine metabolism by mediating the degradation of nucleotides. Moreover, heightened glycolysis, worsened glucose intolerance, increased aminotransferase activity, and reduced UA excretion may underlie hyperuricemia in native Tibetans.

## Data availability statement

The datasets used and analyzed during the current study are available from the corresponding author upon reasonable request.

## Ethics statement

The studies involving humans were approved by Chinese PLA General Hospital (approval identifier S2021-016-01). The studies were conducted in accordance with the local legislation and institutional requirements. The participants provided their written informed consent to participate in this study.

## Author contributions

X-WR conceived the project, recruited the populations, and collected samples and clinical data. KC, JW, Z-LY, and TJ helped collect and detect the samples. Q-HZ statistically analyzed and interpreted the data and wrote the draft of the manuscript. All the authors have read and approved the final manuscript.

## Supplementary material

Supplementary Figure 1 The normality test of variables. The biochemistry data in the respective Tibetans and the Hans from the three groups, as well as data in the blood routine test in Group C were analyzed for normality by Shapiro-Wilk test.

Supplementary Figure 2 Comparisons of the biomarkers for heart failure between the Hans and the Tibetans with normal or high uric acid (UA) levels in Group A. **P*<0.05, normal UA *vs.* high UA.
