# Supplementary figures and images for: Distinctive biochemistry profiles associated with hyperuricemia between Tibetans and Hans in China
Source: Front Endocrinol (Lausanne). 2023 Nov 28;14:1229659. doi: 10.3389/fendo.2023.1229659 (PMC10715267; doi:10.3389/fendo.2023.1229659)

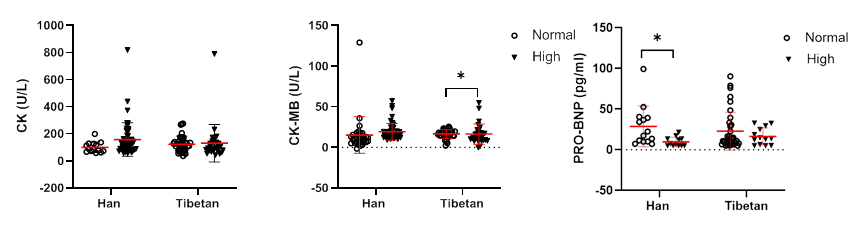

Supplement: Supplementary Figure 1 — The normality test of variables. The biochemistry data in the respective Tibetans and the Hans from the three groups, as well as data in the blood routine test in Group C were analyzed for normality by Shapiro-Wilk test. [file Image_1.tif]

## Slide 1
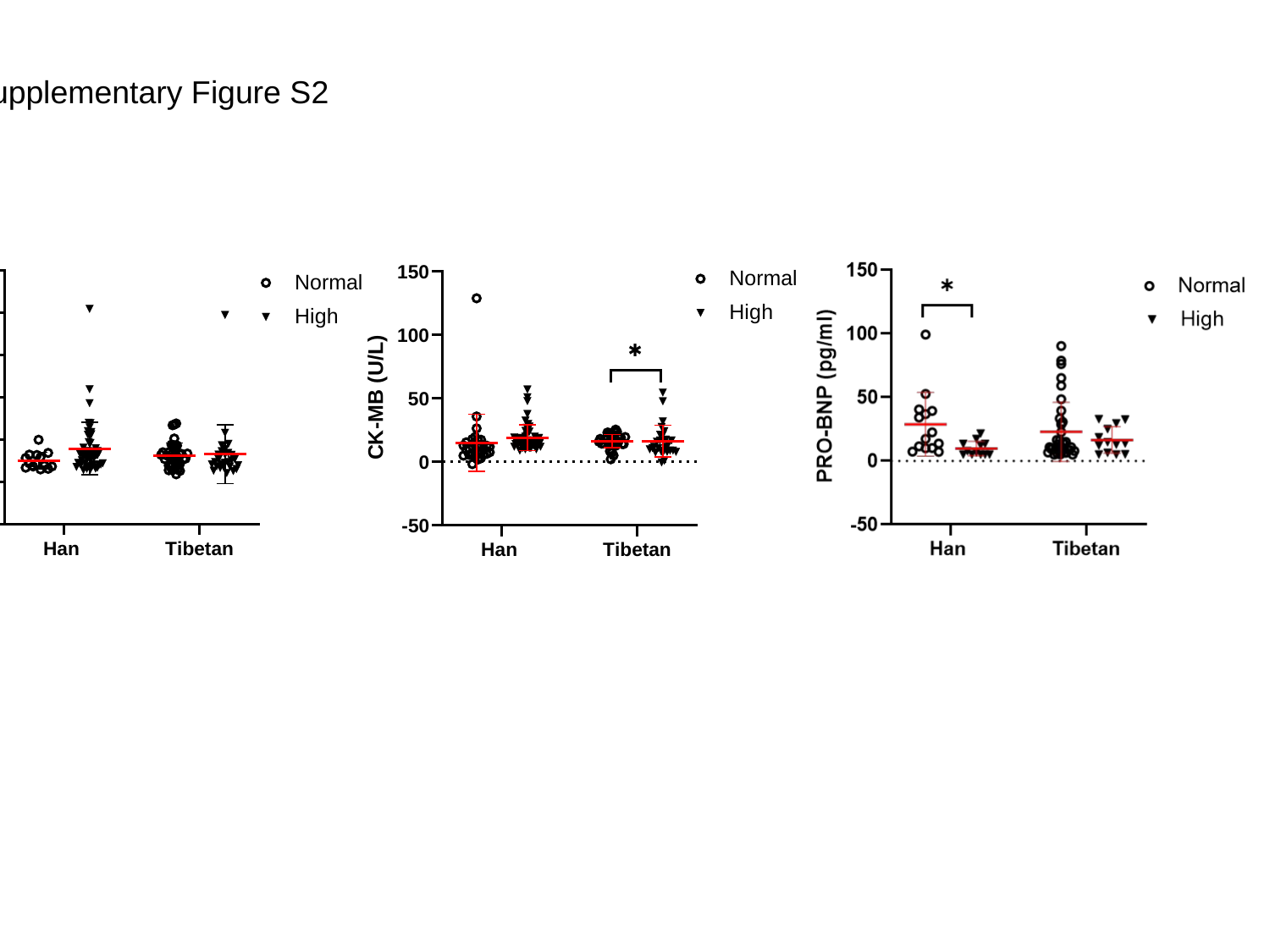

Supplementary Figure S2

Supplement: Supplementary Figure 2 — Comparisons of the biomarkers for heart failure between the Hans and the Tibetans with normal or high uric acid (UA) levels in Group A. *P<0.05, normal UA vs. high UA. [file Presentation_2.pptx]
